# How do environmental stressors influence migration? A meta-regression analysis of environmental migration literature

**DOI:** 10.4054/demres.2024.50.2

**Published:** 2024-01-05

**Authors:** Shuai Zhou, Guangqing Chi

**Affiliations:** 1 Department of Global Development, Cornell University, Ithaca, New York, USA. Department of Agricultural Economics, Sociology, and Education, Population Research Institute, and Social Science Research Institute, Pennsylvania State University, University Park, Pennsylvania, USA.; 2 Department of Agricultural Economics, Sociology, and Education, Population Research Institute, and Social Science Research Institute, Pennsylvania State University, University Park, Pennsylvania, USA.

## Abstract

**BACKGROUND:**

The amount of literature on environmental migration is increasing. However, existing studies exhibit contradictory results. A systematic synthesis of the environment–migration relationship is much needed.

**OBJECTIVE:**

This study summarizes research findings, calculates the effect sizes of environmental stressors, identifies publication bias, and investigates heterogeneous environmental effects on migration.

**METHODS:**

We collected 3,380 estimates from 128 studies published between 2000 and 2020 to explore the environment–migration relationship and performed weighted instrumental variable regression to unveil the heterogeneous environmental effects on out- and net migration.

**RESULTS:**

The majority of environmental stressors were not important predictors of out- and net migration. Among the results showing environmental impacts on migration, 58% and 68% reported that environmental stressors increased out- and net migration, respectively, while 58% reported that environmental stressors decreased in-migration. The overall environmental impact on migration was small; however, disaster-related stressors showed a medium effect, and rapid-onset stressors had a stronger impact than slow-onset ones. Multivariate meta-regression analyses demonstrated that environmental stressors were more likely to trigger internal migration than international migration and that developed countries were less likely to experience out-migration. Rapid-onset environmental stressors did not increase out-migration but played an important role in decreasing net migration toward environmentally stressed areas. Meanwhile, we also found a publication bias toward studies showing a positive relationship between environmental stressors and migration in the previous environmental migration literature.

**CONCLUSIONS:**

Environmental stressors may affect migration; however, the environmental effect depends on migration measurements, environmental stressors’ forces and rapidity, and the context in which migration takes place.

**CONTRIBUTION:**

This study contributes to migration studies by synthesizing and validating the environment–migration relationship and enhancing our understanding of how and under what circumstances environmental stressors may affect migration.

## 1. Introduction

Environmental changes have been influencing migration all over the world (Black et al. 2011; Piguet, Kaenzig, and Guélat 2018). Previous studies have estimated that environmental changes could create hundreds of millions of environmental migrants globally by 2050, although such estimates should be interpreted with caution because of the varying degrees of empirical rigor and environmental change scenarios (Gemenne 2011; Kaczan and Orgill-Meyer 2020). Numerous studies have explored the environmental impact on migration, but conflicting findings, arising from the complexity of migration and variations in measurement approaches, datasets, and analytical methods, make it challenging to draw definitive conclusions (Hunter, Luna, and Norton 2015).

Although several systematic reviews exist, they are either from a methodological standpoint (Piguet 2010), conducted at certain geographic scales (e.g., the country level; see Hoffmann et al. 2020), or focused on specific places, such as African countries (Borderon et al. 2019) or Sahelian countries (Neumann and Hermans 2017). Several studies have explored the impact of study-specific characteristics on the environment–migration relationship using regression approaches. For instance, Beine and Jeusette (2019) analyzed 51 papers from the environmental migration literature and found that measurements of migration and environmental stressors, statistical approaches, and the specification of regression models are important factors that contribute to the heterogeneous findings in previous environmental migration literature. Similarly, Šedová, Čizmaziová, and Cook (2021) conducted meta-regressions using 3,625 estimates of the environment–migration relationship from 116 papers, discovering that extreme temperatures and drought drive migration while sudden-onset events do not and that environmental migration is more common in middle-income countries, with women and those in low-income countries facing a higher risk of being trapped in affected areas.

One of the weaknesses of the meta-analysis studies is that they do not quantify comparable effect sizes between environment and migration regarding issues of varying measurements and analytical approaches. Using meta-regression analysis on comparable effect sizes from what we believe to be the most inclusive literature set (3,380 estimates^3^ from 128 empirical studies), this paper fills the knowledge gap and enhances our understanding of how and under what circumstances environmental stressors may affect migration. We answer three questions: What are the major research findings regarding the relationship between environment and migration in the literature? What are the effect sizes among different environmental stressors on migration? What characteristics affect the effect size of environmental stressors on migration, particularly out- and net migration?^4^ The analyses are conducted at the environmental stressor level.

The paper is structured as follows. First we review the existing environmental migration literature and discuss the heterogeneities in the findings. Then we introduce the meta-regression approach and the process and criteria for selecting literature, followed by the coding strategy and analytical approach. Next we describe the current trend, average effect sizes, and publication bias. We then apply multivariate meta-regression to examine the heterogeneous relationship between environmental stressors and migration with a focus on out- and net migration. We conclude with discussions on the results and contributions, and we propose future research directions.

## 2. Prior environmental migration research

### 2.1 Measurements and environmental migration

The concept of migration is ambiguous, and determining whether someone is a migrant depends on arbitrary criteria, such as the distance and time period covered by the geographic movement. In this study, to account for the diversity of definitions of migration in environmental migration studies worldwide, we broadly define migration as the movement of a person or a group of people across specific geographic or administrative boundaries (e.g., county, state, province, or regional boundaries) within a certain time frame (e.g., nonspecified short term, one year, or five years).

Based on our definition, we identified several migration measures and found both agreements and disagreements regarding the environmental influence on distinct migration patterns. First, many studies found that people turn to out-migration when facing environmental pressures, such as rainfall shortage (Gray and Mueller 2012) and resource scarcity (Massey, Axinn, and Ghimire 2010). However, environmental stressors sometimes rapidly deplete the resources for migration or increase labor demands for reconstruction, which then decreases out-migration (Nawrotzki and DeWaard 2016). Second, migration responses to environmental stressors differ in their durations. Henry et al. (2004) tested the impact of rainfall deficits on the duration of migration using data from Burkina Faso; results suggested that rainfall deficits tend to increase long-term migration to rural areas while suppressing short-term moves to distant destinations. Third, regarding migration across national borders, some studies found evidence that environmental stressors increase internal rather than international migration (Henry et al. 2004). Nevertheless, exceptions exist, especially among geographically close countries. For example, using census data and precipitation data from dry areas of Mexico, Nawrotzki, Riosmena, and Hunter (2013) found that rainfall deficits increased U.S.-bound international migration. Indeed, a recent study has shown that most international migrations occurred within continents and between neighboring countries (Vestby, Tollefsen, and Buhaug 2022). This trend is likely attributed to geographical proximity, relatively lower migration costs, preexisting migration networks, and increased opportunities for obtaining visas for movement.

Environmental stressors are diverse and place-specific, and their effects on migration also differ. Rapid- and slow-onset environmental stressors are the commonly used distinctions in the literature (Koubi et al. 2016). Rapid-onset extreme disasters such as floods, tsunamis, and hurricanes are closely associated with out-migration, but the migration tends to be short-distance and is usually followed by return migration (Black et al. 2011; Groen and Polivka 2010; Warner et al. 2010). Slow-onset environmental changes such as drought, desertification, and land degradation tend to incur short-distance and temporary migration, but migrants’ main aim is to diversify livelihood strategies rather than escape from environmental stressors. Meanwhile, migration responses to both slow-onset environmental stressors (Findlay 2011; Fussell, Hunter, and Gray 2014) and rapidly evolving environmental disasters (Smith and McCarty 2009; Thiede and Brown 2013) are often selective on sociodemographic characteristics, with socially advantageous groups (identified by education level and ethnicity) generally more likely to move under environmental pressures (Henry, Schoumaker, and Beauchemin 2004). Besides the varying effects between rapid- and slow-onset environmental stressors, classifying environmental stressors by their forces and typologies (e.g., disaster, temperature and precipitation variability, and environmental stressors by rapidity)^5^ also shows heterogeneous environmental impacts on migration, particularly across time, space, and place (Leyk et al. 2012; Vestby, Tollefsen, and Buhaug 2022). With a focus on environmental forces, policy responses, and mobility patterns of the affected population and using data from 321 published case studies on environmentally induced displacement, Kaenzig and Piguet (2021) provided a typology framework for understanding the complex and varied ways in which environmental change, particularly slow-onset environmental degradation, can lead to varying displacement and migration patterns.

It is reasonable to expect heterogeneous results across different classifications of environmental stressors. Nevertheless, the same environmental stressors may affect migration differently, even in the same place at the same time, which is often seen in different sociodemographic groups. For example, Gray and Mueller (2012) found that drought in rural Ethiopia increased men’s labor migration while suppressing women’s marriage-related migration.

### 2.2 Methodological approaches and environmental migration

From a quantitative perspective, previous studies employed multivariate regression, multilevel analysis, agent-based modeling (ABM), and spatial techniques to explore the environment–migration relationship (Fussell, Hunter, and Gray 2014; Safra de Campos, Bell, and Charles-Edwards 2017; Miller and Vu 2021). Below we highlight their applications and findings.

Multivariate regression is a commonly used method in exploring the environment–migration relationship. Studies employing multivariate regression often utilize ordinary least squares (OLS), logistic, and event history methods, depending on whether they treat the outcome as continuous variables, binary variables, or events. Our general observation is that the results from multivariate regression vary across time and place. For instance, while drought was found to be negatively associated with migration in the 1980s in Mali (Findley 1994), it increased international migration from Mexico to the United States (Nawrotzki, Riosmena, and Hunter 2013) and internal migration in Ethiopia (Gray and Mueller 2012). These regression models often encompass various levels of analysis. At the micro-level, they investigate individual decisions based on survey data, focusing on specific environmental migration patterns. At the macro-level, these regression models examine migration flows, offering a broader perspective by exploring aggregated trends and patterns across geographical spaces. Employing a multilevel approach is ideal, as we discuss below, as it allows exploration and capture of the interplay between various levels, such as individual, contextual, and spatial factors. This approach provides a more comprehensive understanding of complex phenomena and their relationships, such as the environment–migration linkage.

Although cluster-robust standard errors (Cameron and Miller 2015) can be applied to account for clustering in the data in multivariate analyses, a common scenario in the migration literature, studies have shown that the multilevel method is a better approach because it recognizes data hierarchies and can disentangle the effect of each level (Cheah 2009; Piguet 2010; Zolnik 2009). This is particularly important for environmental migration studies because migration decision-making is affected not only by individual and household characteristics but also by contextual characteristics such as local climatic conditions. Through comparisons between multivariate and multilevel methods, Chi and Voss (2005) empirically showed that the multilevel approach provides advantages in the following ways. First, it allows researchers to combine heterogeneous variables at the aggregate level into one model; second, it can estimate the reliability of coefficients for level-1 variables in a multi-level hierarchy; and third, it potentially avoids debates about ecological and atomistic fallacies. The study also confirmed that environmental amenities decreased out-migration in Wisconsin in the United States.

The ABM approach, which can account for the agency of autonomous entities when making migration decisions, is useful for capturing dynamic, interactive, and nonlinear relationships within the process of environmental migration decision-making (Hunter, Luna, and Norton 2015; Piguet 2010). ABM could also serve as a tool for theory-building through exploring theoretical mechanisms in complex social phenomena (Smith and Conrey 2007). Using ABM, Entwisle, Williams, and Verdery (2020) applied different rules derived from empirical surveys and ethnographic data to guide their constructed agents, including individuals, land parcels, and households, to investigate the dynamic and interactive pathways through which environmental stressors might affect migration in Nang Rong, Thailand. The simulation results showed that environmental stressors had little impact on out-migration but had a markedly negative effect on return migration.

Spatial analysis of migration has gained popularity over the last few decades. Although spatial regression may use similar estimation methods, such as maximum likelihood, it can identify and account for spatial effects embedded in the migration process, such as spatial dependence and spatial heterogeneity. Using spatial regressions, Saldaña-Zorrilla and Sandberg (2009) found that high disaster frequency increased out-migration at the municipality level in Mexico. However, spatial regressions inevitably encounter ecological fallacy issues, where results from one spatial level may not hold at other spatial levels (Leyk et al. 2012), resulting in different environment–migration relationships at different spatial levels.

### 2.3 Place-specific characteristics and environmental migration

Place-specific characteristics also play a role in the environment–migration relationship. Places with different stages of economic growth and social networks may exhibit different environmental migration patterns. Studies found that in developing countries such as Ghana (Codjoe et al. 2017) and Bangladesh (Bohra-Mishra, Oppenheimer, and Hsiang 2014), environmental migration was driven primarily by economics; environmental stressors played a secondary role. Nawrotzki and DeWaard (2018) and Logan, Issar, and Xu (2016) showed that people from developing areas and socioeconomically vulnerable backgrounds were less mobile under environmental pressures. Migration networks also influence environmental migration. Using data on global immigration to the United States and hurricane indices, Mahajan and Yang (2020) found that the effect of hurricanes on immigration was magnified by existing immigration networks between origin countries and the United States.

## 3. Methods

### 3.1 Meta-analysis and the application of partial correlation coefficient

Meta-analysis is a powerful method to establish evidence-based knowledge by reviewing numerous studies addressing the same research question and comparing their results (Gheasi et al. 2019; Gurevitch et al. 2018). This approach provides a high level of evidence in scientific research (Berlin and Golub 2014).

One of the purposes of meta-analysis is to explore how study-specific characteristics lead to different effect sizes – a quantitative measure of the impact of independent variables on the dependent variable. Effect sizes typically fall into three families, r-based, d-based, and odds-based,^6^ among which r-based effect sizes are preferred in the social sciences because parameter estimates in social science studies are often based on the correlation between independent and dependent variables (Ringquist 2015). Cohen’s d and Hedges’s g can also be used to estimate effect sizes in experimental settings (Fritz, Morris, and Richler 2012). However, in social sciences such as demography and sociology, experimental studies are rare, and the measures of a phenomenon can vary greatly. Accordingly, the methodological approaches and results vary. These situations cause difficulties in retrieving and calculating comparable effect sizes for meta-analysis.

The partial correlation coefficient (PCC) is an appropriate r-based effect size measure to deal with these difficulties (Oczkowski and Doucouliagos 2014). A PCC is a standardized effect size that is adjusted for all other covariates in the regression models; it represents the strength and direction of the association between the dependent variable and independent variables regardless of the metrics used to measure them or the estimation methods (Ogundari and Bolarinwa 2019). Simply put, a PCC is analogous to Pearson’s correlation R, which represents the strength and direction of a linear association between two variables. The formulas for calculating the PCC and associated standard error are as follows (Ringquist 2015):

(1) $$PCC_{ij}=\frac{t_{ij}}{\sqrt{t_{ij}^{\;\;2}+df_{ij}}}$$

(2) $$SE_{PCC_{ij}}=\sqrt{\frac{1-{PCC}_{ij}^{\;\;2}}{df_{ij}}}$$

where $PCC_{ij}$ in Equation (1) denotes the PCC of the $j_{th}$ environmental factor in the $i_{th}$ study and $t_{ij}$ and $df_{ij}$ are the t-statistic and degrees of freedom, respectively. $SE_{PCC_{ij}}$ in Equation (2) is the standard error of $PCC_{ij}$. The PCC ranges from −1 to 1, where negative and positive values represent negative and positive associations, respectively. There are two advantages to using PCC. First, calculating PCC requires only the t-value and the degrees of freedom, which are widely available in the reported results of environmental migration studies. Second, because PCC is a standardized effect size, it is comparable among studies regardless of their measurements and analytical approaches.

Although promising in harmonizing and synthesizing results from different datasets, measurements, and analytical approaches, a meta-analysis with PCC comes with limitations. First, constructing PCC requires at least correlation coefficients or regression results. Therefore studies that report only summary statistics are excluded, which limits the sample sizes in the meta-analysis. Second, different disciplines, journals, and statistical programs report different statistics, creating difficulties in constructing PCC. For example, some regression tables may report only coefficients and significance levels, indicated by asterisks or other symbols. To include such studies, one compromise is to obtain the lower-bound t-statistic first and then proceed with the calculation of PCC. The inclusion of studies without reporting suitable statistics for constructing PCC is a conservative approach and potentially results in an underestimated relationship between environmental factors and migration, as the true effect sizes are likely higher than those obtained using low-bound t-statistics.^7^ Third, meta-analysis depends on an arbitrary p-value threshold (p<0.05 in this study) to establish statistical significance for identifying publication bias and performing other p-value-related analyses. However, the scientific community has acknowledged that relying solely on the p-value may not provide sufficient evidence for making valid and meaningful conclusions (Siegfried 2010; Wasserstein and Lazar 2016).

### 3.2 Literature search and exclusion criteria

In most meta-analysis studies, collecting literature involves manually searching journals or electronic databases using a series of combinations of keywords. In the environmental migration area, Piguet and colleagues (2018) have been maintaining the CliMig database, a comprehensive and updated list of publications focusing on environmental migration. To ensure comprehensiveness, CliMig tracks and collects a wide range of topics related to environmental migration and includes journal articles, books, and gray literature such as reports, proceedings, and working papers. The quality and exhaustiveness of CliMig have been previously confirmed by comparing search results with those from Scopus and the Web of Science (Borderon et al. 2019; Piguet, Kaenzig, and Guélat 2018). On the basis of this proven depth of coverage, we extracted the literature for our analysis from the CliMig database. As of August 25, 2020, there were 1,412 publications in CliMig. To prevent incompleteness and bias, we referenced an additional seven published works (Table A-1) that synthesized environmental migration literature and included 351 additional empirical studies that were not included in the CliMig database. These two approaches provided us with 1,497 unique publications from 1945 to 2020.

**Figure 1:**
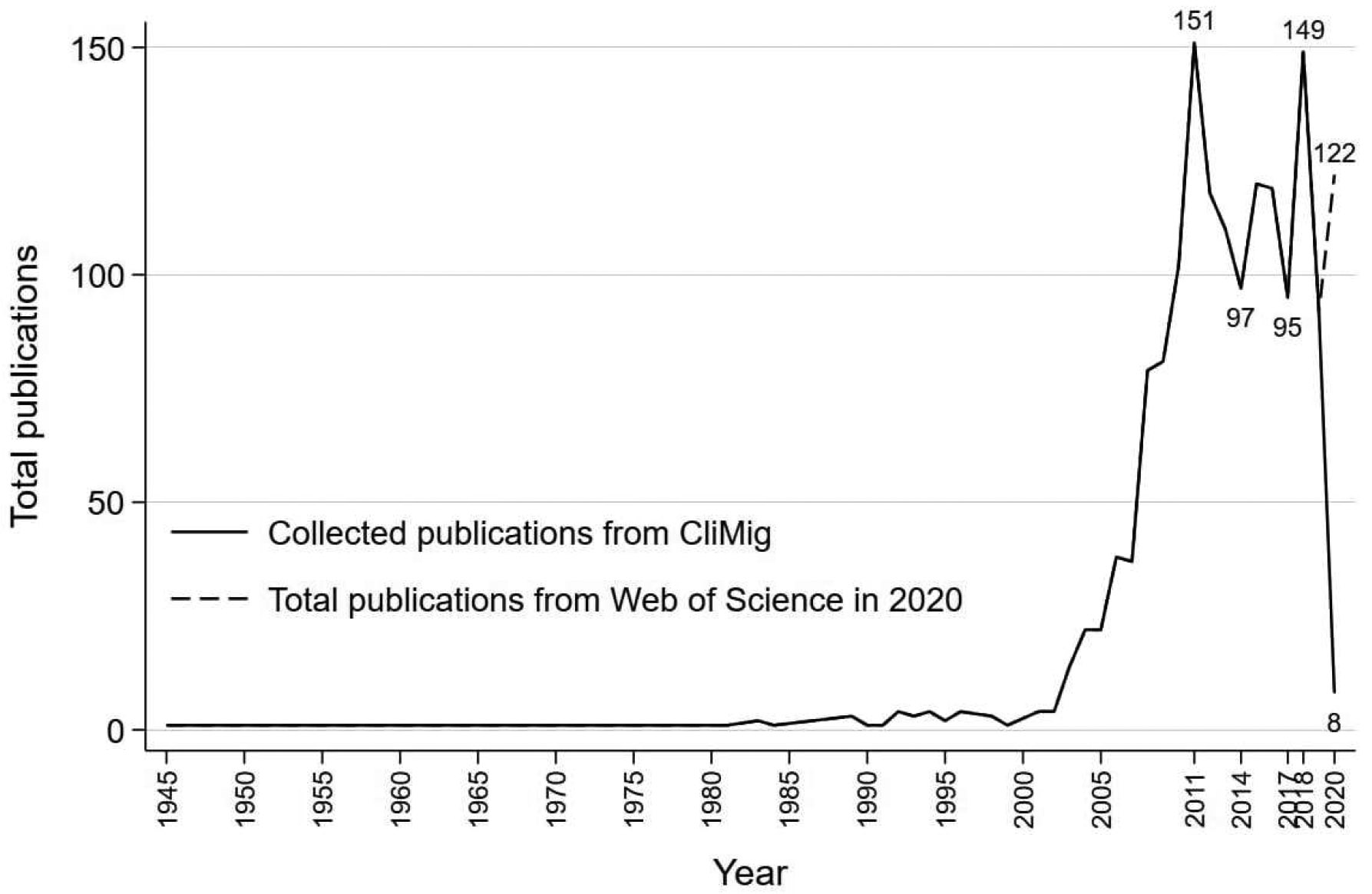
Publications on environmental migration from CliMig database and literature searches from seven published systematic reviews on the environmental migration literature (n = 1,497), 1945–2020 *Note*: The solid line represents collected publications from the CliMig database across years. The dashed line represents publications on environmental migration from the Web of Science in 2020. We used the following criteria to filter the studies: topic = “environmental migration”; publication years = 2020; research areas = “geography” + “demography” + “sociology” + “anthropology” + “social sciences other topics.” The Web of Science gives 122 publications based on the filter criteria.

Figure 1 shows the distribution of the publications. From 1945 through 2000, environmental migration studies were sporadic. From 2000 onward, there was a rapid increase, reaching a peak in 2011. After 2011 there was a decrease, and then a fluctuation until 2018, when the numbers rebounded. The peak around 2011 may have been stimulated by the awarding of the Nobel Peace Prize to the 2007 Intergovernmental Panel on Climate Change (IPCC) report (Piguet, Kaenzig, and Guélat 2018). The fluctuation of publications in the environmental migration field between 2011 and 2018 might reflect the public’s skepticism toward climate change and the availability of funding for climate-related research around the globe (Whitmarsh and Capstick 2018). Because our literature collection ended in August 2020, publications from the rest of 2020 are missing from our dataset. Therefore there is a drop in collected publications in 2020.

**Figure 2:**
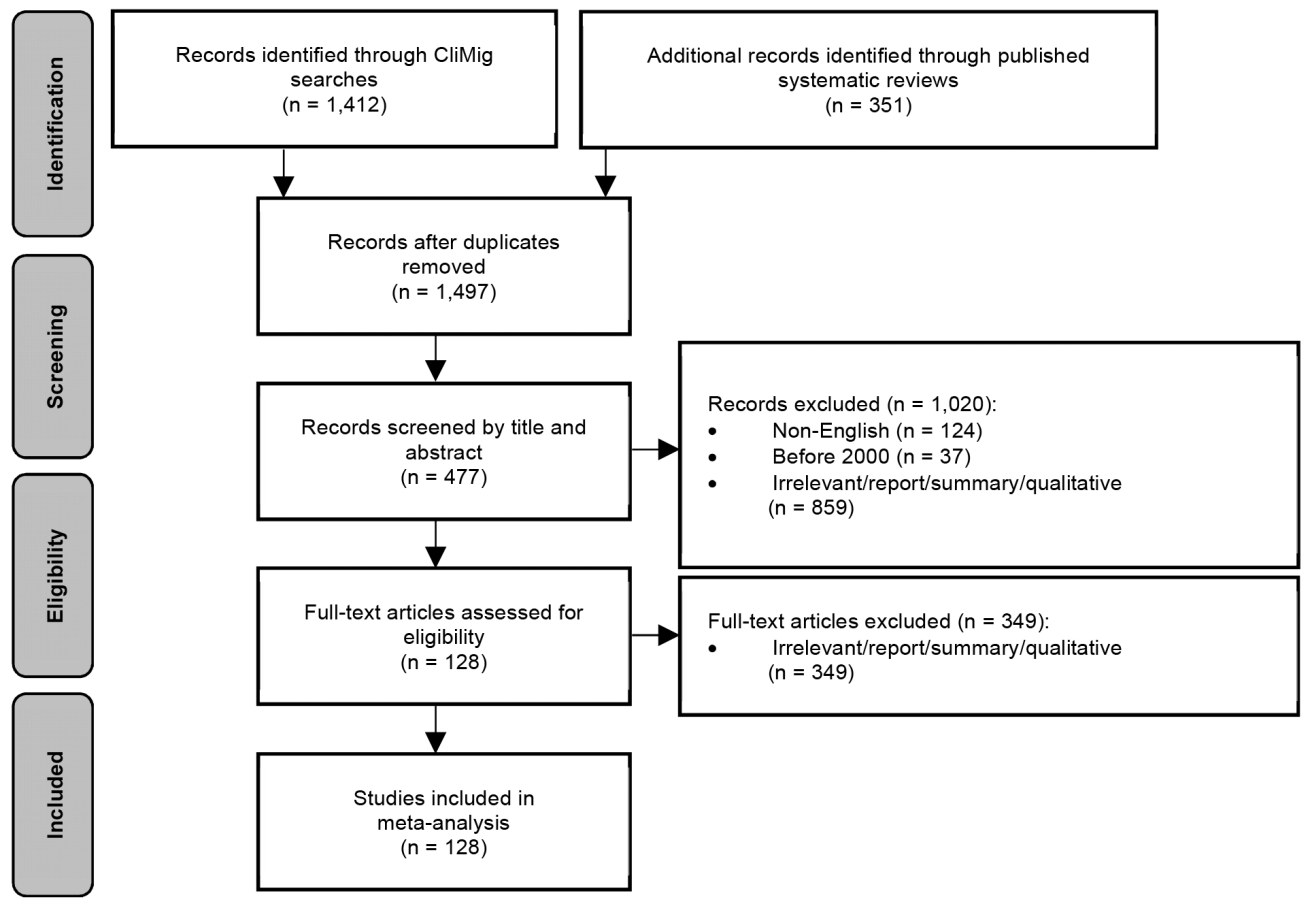
Modified PRISMA flow diagram explaining the process of selecting literature for the meta-analysis

Figure 2 shows a Modified Preferred Reporting Items for Systematic Reviews and Meta-Analysis (PRISMA) diagram (Moher et al. 2009) explaining our strategies in filtering the literature. We provide the detailed literature filtering processes and a full list of selected literature in Appendices B and C, respectively.

### 3.3 Coding strategy

Table 1 shows coding strategies for the chosen characteristics from the collected studies.

#### Coding environmental stressors.

Measuring environmental stressors remains an open and challenging issue because of the complexity and interdependency of the elements within the ecological and climatic system. Building on previous scholarly endeavors in categorizing environmental stressors (e.g., Kaenzig and Piguet 2021; Lonergan 1998; Stojanov et al. 2014), this study differentiates environmental stressors according to their forces (Henry et al. 2004) and velocities (Kniveton et al. 2008), the two common dimensions of environmental stressors. Table 2 shows the original environmental stressors from the selected studies and our coding schemes. Depending on the forces, we categorized environmental stressors into the following groups:

1. Disaster-related stressors. This category refers to environmental events occurring over a short period of time and causing devastating consequences to people and their properties, including earthquakes, bush fires, hurricanes, storms, tsunamis, and volcanic eruptions.^8^
2. Precipitation-related stressors. This category refers to environmental measures that are related to precipitation, rainfall, and monsoons, which are common environmental stressors examined in previous environmental migration literature. Specifically, this category includes precipitation and rainfall measured in terms of absolute value and frequency, monsoon delay, drought, and flood.^9^
3. Temperature-related stressors. This category refers to temperature-related measures in the forms of absolute values, extremes, and anomalies. One publication (Afifi and Warner 2008) in the selected studies focused on sea level rise. For reasons of simplicity, we included sea level rise in temperature-related stressors because it results primarily from global warming.
4. Land-related stressors. This category refers to environmental measures that affect land and soil, including damaged land, deforestation, desertification, land/soil erosion and degradation, land quality, landslides, soil pollution, and soil salinization.
5. Loss-related stressors. This category refers to environmental measures related to crop/property loss. Some studies (e.g., Feng, Oppenheimer, and Schlenker 2012) did not explicitly specify this type of environmental stressor; rather they used crop/property as a proxy for environmental impact on migration. This category includes losses in the forms of crop failure, livestock loss, and property damage.^10^
6. Others. This category refers to general measures of climate, weather, and environmental conditions that cannot be meaningfully included in the above categories, including air pollution, climate impact, environmental and weather condition, humidity, wind, El Niño, La Niña, the Normalized Difference Vegetation Index (NDVI), and the Standardized Precipitation-Evapotranspiration Index (SPEI).

Apart from the above classification by environmental forces, we created another variable to classify environmental stressors by velocity and grouped them into rapid- and slow-onset stressors:

1. Rapid-onset stressors. Rapid-onset environment stressors are environmental measures that evolve rapidly and threaten populations and properties in a short period of time. This category includes environmental stressors in the disaster-related stressors category defined above, as well as flooding.
2. Slow-onset stressors. Slow-onset environmental stressors are environmental measures that evolve in a relatively slow-acting process. This category includes remaining environmental stressors that do not belong to the rapid-onset stressor classification defined above.

It is difficult to meaningfully separate environmental stressors by their forces because they are interrelated. For instance, disasters such as hurricanes also bring heavy rainfall, which may cause floods, landslides, and crop and property losses. Also, slow- and rapid-onset environmental stressors may not be separable because they are situated in a spectrum without a clear-cut point of differentiation. The two classifications we applied to code environmental stressors create mutually exclusive categories by their forces and velocities. These serve our research questions on the one hand and are in line with the current literature on the other hand.

#### Coding migration.

Diverse methods of migration coding signify varying levels of human agency, purposes, directions, and temporal attributes of movement, significantly influencing the estimated effects of environmental factors on migration (Vestby, Tollefsen, and Buhaug 2022). We focused on migration directions and the assessment of whether such movements cross national borders under environmental change. Thus we categorized migration into out-, in-, and net migration based on movement direction while also distinguishing between internal and international migration based on border-crossing status. Net migration was included because some of the studies defined migration as net population change (e.g., Logan, Issar, and Xu 2016) and net migration rate (e.g., Gutmann et al. 2005).

#### Coding study characteristics.

This includes information about where, when, and how the study was conducted. We coded the following characteristics of each study:

1. Study region: Depending on the study regions and their economic development, we classified the previous studies as OECD countries, non-OECD countries, and global if the studies involved international bilateral migration flows (e.g., Maurel and Tuccio 2016).
2. Inclusion of control variables: It is widely acknowledged that environmental stressors take effect in combination with other socioeconomic, demographic, cultural, and political covariates. Knowing this, we differentiated previous studies depending on whether they included those variables.
3. Dataset time period: On the basis of the time period for which the datasets were collected, we divided the dataset time period into dummy variables indicating whether they were collected in the 1970s (including 0.01% of the total estimates from data collected before 1970), 1980s, 1990s, 2000s, or 2010s. For panel data, we used the midpoint to determine each study’s time period.

### 3.4 Analytical approach

In the collected literature, each study may report different statistics or regression results. While coding, we encountered four scenarios, for which we utilized different strategies to calculate the PCC and its standard error:

1. If the study reported t-statistics, we used Equations (1) and (2) to calculate the PCC and associated standard error.
2. If the study reported standardized coefficients, we followed Ringquist’s (2015) suggestion to treat the standardized coefficient as the PCC and calculated the associated standard error using Equation (2).
3. If the study reported coefficients and standard errors but did not report t-statistics, we obtained the t-statistics first by dividing the coefficient by the standard error and then used Equations (1) and (2) to calculate the PCC and associated standard error.
4. If the study reported only coefficients and significance levels designated by asterisks or other symbols, we set the t-statistics as the value of the two-tailed t at the symbol threshold level and given degrees of freedom (Ringquist 2015). The PCC and associated standard error were then calculated using Equations (1) and (2). It should be noted that this scenario (22% of the estimates) gives the lower-bound t-statistic. The PCC is therefore underestimated.

We noted that some studies included interaction and quadratic terms (e.g., Gutmann et al. 2005). The presence of interaction and quadratic terms makes the calculation and interpretation of PCC complicated. Following Šedová, Čizmaziová, and Cook’s (2021) approach, we excluded the estimates of interaction and quadratic terms from this study. The current coding strategy already gave large numbers of estimates, which guaranteed sufficient statistical power. The exclusion of interaction and quadratic terms should not remarkably affect the results.

After obtaining the PCC and associated standard error, we estimated the overall weighted average PCC and the weighted average PCC by environmental force and velocity across out-, in-, and net migration using the following weight:

(3) $$w_{i}=\frac{1}{{{SE}_{PCCi}}^{2}}$$

where $w_{i}$ is a precision-based weight for each estimate; it gives more weight to estimates with less variance while down-weighting highly uncertain estimates. According to Marín-Martínez and Sánchez-Meca (2010), the precision-based weight yields more accurate estimates of the effect size than other weighting methods, such as the sample size–based weight.

We then applied meta-regression analysis to investigate the heterogeneous environment–migration relationship in the selected literature. The meta-regression formula is as follows:

(4) $$PCC_{ij}=\beta_{0}+\beta_{1}Env_{ij}+\beta_{2}Mig_{ij}+\beta_{3}Study_{i}+\beta_{4}se_{ij}+\varepsilon_{ij}$$

where $PCC_{ij}$ represents the PCC between the $j_{th}$ environmental stressors and migration in the $i_{th}$ study; $Env_{ij}$ and $Mig_{ij}$ are environmental and migration measures, respectively; $Study_{i}$ represents the study-specific characteristics; and ε represents the error term. Note that PCC is measured at the environmental stressor level, not at the study level. Since each study has multiple environmental stressors, the observations (3,380) from Equation (4) are much greater than the number of studies (128) included in the meta-analysis.

The estimates from the above regression may be biased because of possible omitted variables (perceptions of environmental changes and adaptation abilities) and selection issues when filtering the environmental migration literature. To correct for these issues, we followed previous studies (e.g., Cazachevici, Havranek, and Horvath 2020; Havranek, Horvath, and Zeynalov 2016) and used the inverse of the square root of the degrees of freedom to instrument for the standard error because it is closely related to the standard error but has less to do with the error terms in Equation (4). We assessed the necessary conditions (relevance and exogeneity) for the instrument and present the results below. To adjust for estimation precision, we applied the precision-based weight in Equation (3) in the meta-regression analysis.

## 4. Descriptive analysis

### 4.1 Overall trend in environmental migration studies

Table 3 presents the descriptive statistics; it also reveals the research trend from the distributions of environmental and migration measures and study characteristics. On average, the PCC between environmental stressors and migration is 0.01, with an average standard error of 0.03. The mean standard error of the PCC was triple the size of the mean PCC, indicating the difficulty and uncertainty in quantifying the relationship between environmental stressors and migration. This also means that the true relationship between environmental stressors and migration may be obscured because of the presence of uncertainty. Regarding migration measures, roughly 53% of the estimates focus on international migration and 73% of the estimates focus on out-migration. The environmental stressors are mainly precipitation-related (35%), followed by disaster-related (26%) and temperature-related (24%). By velocity, slow-onset (71%) environmental stressors are the primary foci of the studies. In terms of countries involved, about 47% of the estimates come from studies conducted in non-OECD countries, followed by OECD countries (31%) and global (22%). Moreover, 81% of the estimates come from regressions that include control variables. About 40% of the selected studies use datasets from the 2000s, followed by the 1990s, 1980s, and 2010s; the 1970s saw the fewest datasets collected for environmental migration studies.

Table 4 presents the distribution of the coefficient estimates of the environmental effect on out-, in-, and net migration from the collected studies without distinguishing whether migration crosses national borders. For the 1,521 estimates pertaining to the relationship between environmental stressors on out-migration, a majority of the estimates (62%) show that environmental stressors are not important predictors of migration; among the 916 significant estimates^11^ (p<0.05 unless otherwise specified), 531 (58%) show positive relationships, indicating that environmental stressors tend to increase out-migration. However, environmental stressors are more likely to decrease in-migration, with 58% of the significant results showing negative relationships.

Echoing findings from existing studies, our meta-analysis results explicitly demonstrate that environmental stressors are more likely to trigger out-migration and are less likely to incur in-migration. An unexpected finding is that 68% of significant results show that environmental stressors are associated with an increase in net migration rate, indicating more in-migrants than out-migrants facing environmental pressures. This seemingly counterintuitive finding can be explained by two theoretical approaches to the environment–migration relationship. First, environmental change may result in trapped populations or environmental immobility by adversely affecting financial and/or social capitals, which can prevent people from moving elsewhere (Nawrotzki and DeWaard 2016). Second, this finding may reflect the minimalist view of environmental migration – that is, environmental stressors may play less important or nondeterministic roles relative to socioeconomic drivers (Kaczan and Orgill-Meyer 2020; Stojanov et al. 2014), which can dwarf their effects on out-migration, indirectly leading to an increase in net migration. Moreover, the seemingly counterintuitive results may also stem from the measurement of net migration. By definition, net migration estimates usually refer to the difference between in-migrants and out-migrants from an area during a given time period. The inclusion of both in- and out-migration complicates the environment–migration relationship and makes the reasons for migration decision-making unclear compared to considering in- and out-migration separately (Johnson et al. 2005). Additionally, as noted before, excessive reliance on statistical significance at arbitrary thresholds can obscure the relationship between environmental factors and migration, resulting in misleading findings or interpretations of the environment–migration relationship.

### 4.2 The weighted average PCC

The standard error associated with each PCC provides an estimation of precision and can be utilized to generate weighted average PCCs using weight from Equation (3) to compare the effect sizes of environmental stressors. Table 5 displays the weighted average PCC for environmental stressors and subgroups categorized by their forces and velocities across out-, in-, and net migration.

According to Cohen’s (1988) guidelines, which use 0.3 and 0.5 as thresholds for determining small, medium, and large effect sizes, we found that environmental stressors have an overall small effect on out-, in-, and net migration, with environmental stressors tending to increase out-migration while suppressing in- and net migration.^12^ When considering the environmental effects on migration by forces, disaster-related environmental stressors (along with the others category, which has only four observations) have medium impacts on out-, in-, and net migration. This finding suggest that people may be more pressed to move when facing environmental disasters.

The weighted average PCC by velocity illustrates the opposite results between rapid- and slow-onset environmental stressors. Specifically, rapid-onset environmental stressors have small effects on increasing out-migration and medium effects on decreasing in- and net migration. Slow-onset environmental stressors have small effects on decreasing out-migration and increasing in- and net migration. These differences may indicate the two general migratory responses to environmental changes. First, rapid-onset environmental stressors (e.g., hurricanes and floods) usually leave few choices beyond migration/relocation for the affected population, thus increasing out-migration; second, the affected population facing slow-onset environmental stressors (e.g., land degradation and sea level rise) may adopt adaptive strategies and become resilient, or it may rely on policy interventions to mitigate environmental impacts during the longer-term, evolving processes of slow-onset environmental stressors, leading to a decrease in out-migration. Note that the weighted average PCCs are in essence aggregated summaries of the environment–migration relationship by their forces and velocities and therefore are not directly comparable to the relationship between any other individual environmental stressor measure and migration from individual empirical studies. So the negative average PCC of slow-onset environmental stressors on out-migration does not necessarily contradict previous findings that slow-onset environmental stressors are positively associated with out-migration (e.g., Bernzen, Jenkins, and Braun 2019; Dallmann and Millock 2017).

### 4.3 Publication bias

Publication bias describes a situation where published literature is not systematically representative of the population of completed studies (Rothstein, Sutton, and Borenstein 2005; Stanley 2005). In other words, among all the works done on a specific topic, some were published and others were not – possibly because publishers and editors prefer certain types of results (e.g., a positive relationship between environmental stressors and migration). A contour-enhanced funnel plot (Palmer et al. 2008) is often used to investigate publication bias. It consists of a scatterplot of each estimate’s effect size against its associated standard error and contours of statistical significance. Estimates with less variance are shown at the top. Estimates with high variance are shown at the bottom (Sterne et al. 2011). In the absence of publication bias, the scatter of the estimates shows a symmetrical inverted funnel shape. However, because the primary reason for some studies not being published is the lack of statistical significance (Easterbrook et al. 1991; Loannidis 1998), if estimates are missing in the contours of low significance or insignificance and cause asymmetry in the funnel plot, it is reasonable to attribute the asymmetry to publication bias.

**Figure 3:**
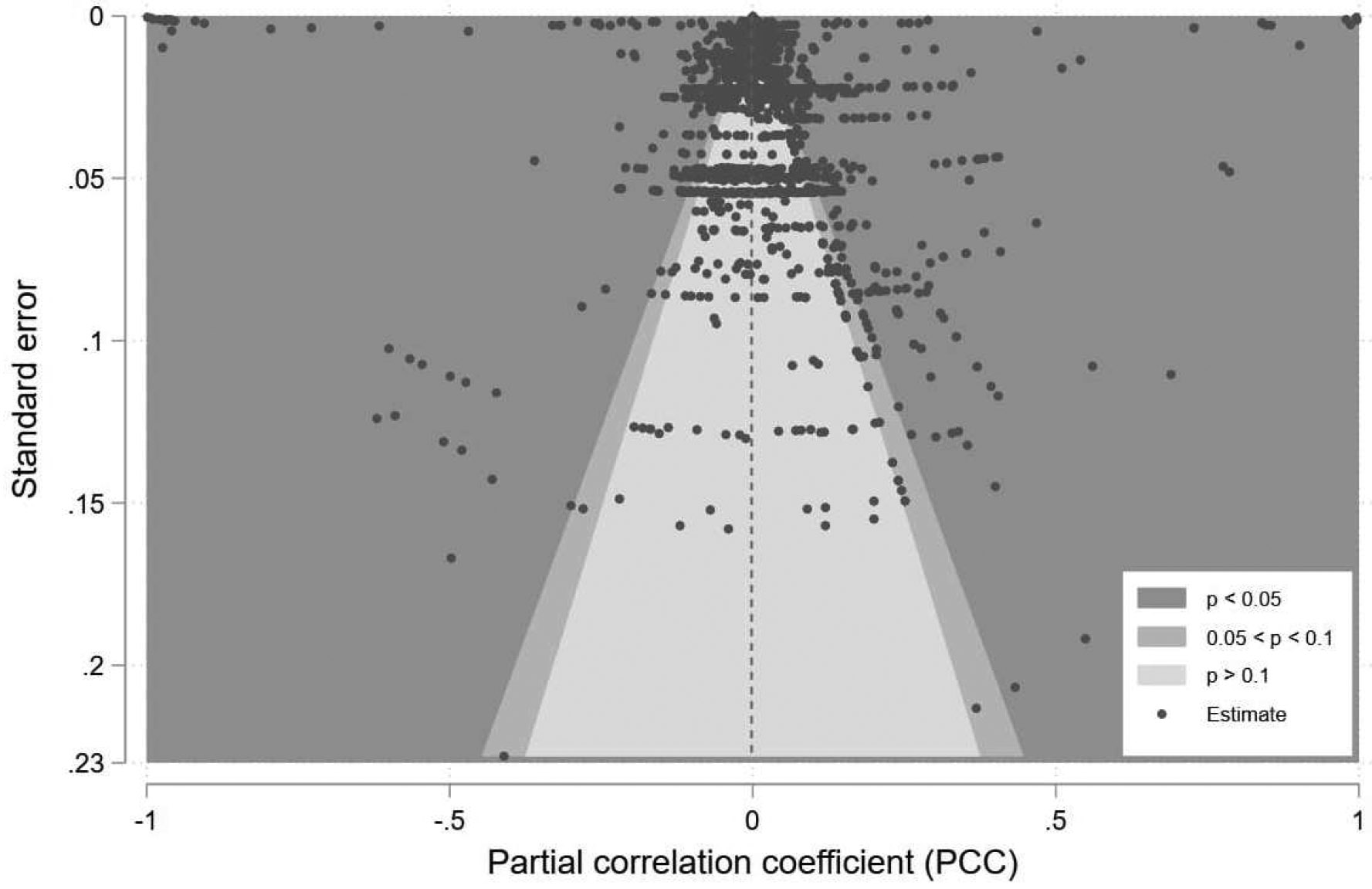
Contour-enhanced funnel plot of partial correlation coeffect (ranges from −0.999 to 0.997) and standard error (ranges from 0.000 to 0.228) for asymmetry test to detect potential publication bias, 2000–2020

Figure 3 shows the contour-enhanced funnel plot for visually detecting publication bias. The distribution of estimates is asymmetric, with missing estimates, including both significant (p<0.05) and insignificant (0.05<p<0.1 and p>0.1) estimates, on the left side. Moreover, the right side is heavier than the left side, particularly for significant estimates. These visualization results indicate a publication bias in the environmental migration literature. We also performed Egger’s test (Egger et al. 1997), a statistical test for funnel plot asymmetry, and the null hypothesis of no publication bias was rejected ($p=1.90\times 10^{\text{-}18}$), thereby confirming the presence of publication bias. Taken together, the contour-enhanced funnel plot and Egger’s test suggest a publication bias toward significant and positive results. In other words, studies with significant and positive findings regarding the environmental impact on migration are more likely to be selected for publication. The presence of publication bias stresses the necessity of applying weight to adjust for such bias in calculating average the PCC and conducting instrumental variable (IV) meta-regression.

## 5. Heterogeneity analysis

We applied multivariate meta-regression to examine the heterogeneous environmental impacts on out- and net migration across various study-specific characteristics. We selected out- and net migration for two reasons. First, out-migration and net migration are two sides of the same coin – namely, out-migration is the most straightforward and intuitive reaction to environmental stressors; net migration represents counterstream migration under environmental stressors. Second, from a data-driven perspective, out-migration and net migration consist of 2,473 (73% of the sample size) and 719 (21% of the sample size) observations, which could provide more statistical power given the relatively larger sample sizes. The sample size of in-migration estimates is too small (188 observations, or 6% of the sample size) to adequately conduct regression analysis. Table 6 shows results from weighted OLS and IV regressions. The F-test from the first-stage regression showed that the instrument is sufficiently correlated with the endogenous regressor and therefore is a valid instrument. The Wooldridge’s score test (Wooldridge 1995) did not reject the null hypothesis that the instrument is exogenous, suggesting the exogeneity of the instrument. Taken together, the two tests provide sound evidence that necessary conditions are met for the chosen instrument.^13^ Meanwhile, the IV regressions show larger R-squared values, suggesting better model fitting; therefore the following interpretations focus on the weighted IV regression results.

For out-migration, we found that disaster-related environmental stressors increase out-migration; other types of environmental stressors do not play important roles. We also found that international migration and OECD countries are negatively associated with out-migration, suggesting that environment-induced out-migration is less likely to cross national borders and less likely to happen in OECD countries. The reduced likelihood of international migration may be attributed to factors such as migration costs and the barriers associated with crossing national borders. Additionally, the decrease in environmental migration observed in OECD countries aligns with the livelihood and adaptation framework that theorizes the relationship between the environment and migration. This framework suggests that places with advanced infrastructure, well-developed economies, and robust capital markets (e.g., OECD countries) are better equipped to adapt to and minimize the impacts of environmental changes on livelihoods, leading to a decrease in migration in the face of environmental challenges.

The results also indicate time effects in the environment–migration relationship. Particularly, datasets collected from the 1990s and 2000s demonstrate a decrease in out-migration, whereas datasets from the 2010s exhibit an increase in out-migration. The decline in out-migration from the earlier periods (the 1990s and 2000s) might be attributed to a lack of recognition and response by governments and the public regarding environmental impacts during those times. Conversely, the observed increase in out-migration in datasets from the 2010s may reflect the following facts: First, environmental change has accelerated more in recent decades (IPCC 2021) and therefore could have more impact on migration. Second, governments in the more recent decades have become more aware of environmental change and its impacts on the population and are more prepared to implement relocation projects in the face of environmental change, resulting in a positive relationship between the dataset time period of the 2010s and migration. Third, the positive relationship may also result from the improved ability in recent decades to collect and analyze large-volume longitudinal and representative environmental and migration data (e.g., census data, the American Community Survey, and the Mexican Migration Project) rather than relying on limited case studies or nonrepresentative data from earlier datasets. When environmental stressors are measured by velocity, the results are similar regarding the effects of international migration, OECD countries, and dataset time periods, indicating strong robustness of model specifications. Moreover, as shown in previous studies (Gray and Mueller 2012; Loebach 2016), rapid-onset environmental stressors are not important predictors of out-migration.^14^

For net migration, the results suggest that disaster-related and rapid-onset environmental stressors decrease net migration. Recall that a decreased net migration means more out-migration than in-migration, so the results from the net migration model generally resonate with those of the out-migration model. The other important predictors of the net migration model include country type, control variables, and dataset time period. Specifically, environmental stressors decrease global net migration. Meanwhile, including control variables decreased the impacts of environmental stressors on net migration, probably because other socioeconomic, demographic, cultural, and political covariates absorbed the environmental effect. Finally, datasets from the 1980s and 1990s showed an increase in net migration, a trend potentially linked to the public’s insufficient recognition of and response to environmental impacts during that era. In other words, the decision-making process for migration might have overlooked environmental considerations, leading to in-migration flows toward regions marked by desirable socioeconomic attributes but also characterized by environmental challenges. Consequently, this phenomenon drove the observed upward trajectory in net migration.

The results from the two models differ in the environmental stressors, which makes sense because they deal with opposite migration responses. The differences between whether or not to include control variables, however, demand further explanations. Again, the main reason may lie in the fact that the net migration measure incorporates both in- and out-migration, which complicates the disentanglement of migration decision-making processes and makes the driving forces differ from those for out-migration (Johnson et al. 2005). Combining in-migration and net migration does not provide better model fitting, indicating that separately analyzing net migration would be the preferable approach.^15^

Methodologically, as previous studies (e.g., Chi and Voss 2005; Zolnik 2009) have done, it is also helpful to consider the hierarchical structure of the data, where individual-level environmental and migration measures are nested in studies at the higher level in modeling the environment–migration relationship. To test the robustness of the results presented in Table 6, we conducted a sensitivity analysis using a two-level regression method. We present the results in Table A-3. The intraclass correlation coefficient (ICC), an indicator that quantifies the proportion of total variation in the outcome that is attributable to between-cluster variation (Sommet and Morselli 2021), suggested that it is necessary to consider the data hierarchy in the out-migration model but not in the net migration model. The rapid-onset environmental stressors, international migration, and OECD countries shown in Table A-3, which are of major interest to this study, show the same coefficient direction as that of Table 6, suggesting robustness of the results. Nevertheless, the difference in the effect of disaster-related environmental stressors between Table A-3 and Table 6 may be due to the substantial ICC values considered in the multilevel model. In conclusion, the sensitivity analyses highlight the importance of considering hierarchical data structures when analyzing the relationship between environmental factors and migration, especially for out-migration driven by environmental impacts.

## 6. Conclusions and discussion

The number of environmental migration studies has been increasing in recent years, resulting in a large body of literature. Previous studies generally showed that the environment–migration relationship is complex and multifaceted (Hugo 2011) and usually not deterministic (Fussell, Hunter, and Gray 2014), which has been demonstrated by the heterogeneous effects of environmental stressors on migration across time and space. From a methodological perspective, the mixed empirical evidence on the environmental impacts of migration can be attributed to a range of choices regarding the conceptualization of core concepts, the selection of methods, and the specification of empirical models (Vestby, Tollefsen, and Buhaug 2022). Given the inconclusiveness in the literature, there is a need to systematically assess those heterogeneous effects to better understand the environment–migration relationship.

Several systematic reviews of the environmental migration literature have been undertaken (Beine and Jeusette 2019; Berlemann and Steinhardt 2017; Borderon et al. 2019; Fussell, Hunter, and Gray 2014; Hoffmann et al. 2020; Hoffmann, Šedová, and Vinke 2021; Kaenzig and Piguet 2021; Neumann and Hermans 2017; Niva et al. 2021; Obokata, Veronis, and McLeman 2014; Piguet 2010; Šedová, Čizmaziová, and Cook 2021; Miller and Vu 2021). However, they did not quantify the environmental impact on different migration measures or explore the heterogeneity in the literature using a comparable effect size across varying research settings. We collected what we believe to be the most current and inclusive empirical studies from the environmental migration literature and conducted a systematic review to explore the research findings, calculate the average effect size, identify publication bias, and investigate the heterogeneous environmental impacts on out- and net migration across different study settings. We contributed to the environmental migration literature by synthesizing and validating the environment–migration relationship and enhancing our understanding of how and under what circumstances environmental stressors may affect migration.

Overall, the findings suggest that environmental stressors are rarely the primary drivers of migration and that environmental impacts on migration are complex and context-dependent, a conclusion that aligns with recent meta-analyses of environmental migration literature conducted at both global (Beine and Jeusette 2019; Hoffmann et al. 2020; Vestby, Tollefsen, and Buhaug 2022) and regional levels (Hoffmann et al. 2022). We found in the previous environmental migration literature that the majority of estimates of environmental stressors are not important predictors of migration. This echoes the minimalist perspective – that environmental factors may not deterministically incur migration; rather, environmental factors tend to be contextual factors that interact with other preexisting drivers of migration (Kaczan and Orgill-Meyer 2020; Stojanov et al. 2014). While the impacts of environmental stressors on migration may be limited or nondeterministic, disparities in their effects across different types and velocities of environmental stressors were identified. Notably, disaster-related and rapid-onset stressors displayed a greater impact on migration compared to other types, as indicated by the weighted average PCC results. As highlighted by Berlemann and Steinhardt (2017), in the absence of coping strategies or adaptive capabilities, migration triggered by sudden climatic changes or natural disasters may be a forced response rather than a voluntary choice.

Furthermore, the contextual dependence of the environment–migration relationship is emphasized through the study-specific effects observed from our empirical models. Specifically, consistent with previous research, we found that rapid-onset environmental stressors do not necessarily increase migration^16^ because such events may quickly deplete the necessary resources for initiating migration (Nawrotzki and DeWaard 2016). When environmental stressors do lead to migration, such movements tend to be internal rather than across national borders, with exceptions observed in geographically contiguous countries such as the United States and Mexico, likely influenced by factors such as geographical proximity, relatively lower migration costs, and preexisting migration networks (Nawrotzki, Riosmena, and Hunter 2013). Non-OECD countries are shown to be more likely to experience environmental migration compared with OECD countries. This finding echoes the livelihood and adaptation framework in explaining environmental migration. Non-OECD countries, owing to their heavy reliance on agricultural and natural resources for their economies and livelihoods, exhibit greater sensitivity to environmental changes and often have limited adaptive capacities to cope with such changes. Consequently, the relationship between the environment and migration in such countries is likely to be stronger compared with that in OECD countries (Hoffmann et al. 2020).

In sum, the findings converge into a coherent story that environmental stressors may affect migration; however, the way they affect migration depends on migration measurements, the force and rapidity of the environmental stressors, and the context in which migration takes place. Although the current evidence, along with findings from similar meta-analyses, has shown that environmental stressors may have limited impacts on migration, this does not mean that their effects can be overlooked. On the contrary, researchers believe that environmental change such as global warming, frequent extreme climatic events, and sea level rise could force millions of people to migrate or compel others to proactively respond to such changes by moving out of environmentally unfriendly places in the near future (Feng, Krueger, and Oppenheimer 2010; Hauer, Evans, and Mishra 2016; Marchiori, Maystadt, and Schumacher 2012).

While we utilized rigorous methods, such as employing weight and IV regression to address estimation accuracy and heterogeneity issues, this meta-analysis comes with three major limitations. First, the results are determined by and dependent on the inclusiveness and representativeness of studies included in the analysis. For instance, this study does not include research written in languages other than English. It is reasonable to assume that the results might vary if non-English studies were included. Second, it is challenging to measure and harmonize different types of migration because of the multiple dimensions involved in migration processes, such as actors, time, distance, and space. Although we differentiated the effect sizes of environmental stressors on out-, in-, and net migration (see Table 5) and modeled their heterogeneous impacts on out- and net migration (see Table 6), we did not capture the entire spectrum of environmental migration and missed long- versus short-distance migration, permanent versus temporary migration, and migration across different sociodemographic groups. Similarly, there are no legitimate ways to group environmental stressors by their forces and velocities because of the interdependency of the climatic system and the barely distinguishable cutoff point in a slow–rapid spectrum. Therefore the average effect sizes and the regression results are also dependent on how we classify those environmental stressors. Third, we relied on an arbitrary p-value threshold (p<0.05) to establish statistical significance for further analyses such as Table 4 and Figure 3. However, it’s important to note that while this is a common practice for determining significance levels and drawing conclusions, depending solely on the p-value could lead to potential errors, misinterpretation, and publication bias (Imbens 2021).

The findings have profound implications for policymakers. First, there is an urgent need to recognize the complex interplay between environmental factors and migration and to incorporate this understanding into migration policies. Specifically, environmental migration policies should consider and address the unique challenges and vulnerabilities faced by affected populations. Second, although non-OECD countries and agriculture-dependent regions are currently more likely to experience environmental migration, environmental change is a global issue that requires international cooperation. This includes investing in sustainable development, improving infrastructure, and implementing early warning systems to mitigate the impacts of environmental hazards.

Future research can advance the field of environmental migration in the following ways. First, the field of environmental migration research offers an excellent opportunity for theory building. It is now time for researchers, especially theorists, to incorporate environmental dimensions into migration theories, particularly in the theorization of forced migration or environmental immobility, two processes that have received relatively less attention within mainstream migration theories (Hunter and Simon 2023; de Sherbinin et al. 2022). Second, it is also crucial for researchers to explore the distinctive pathways and mechanisms through which environmental change manifests in countries and regions with varying levels of resilience to environmental factors. These endeavors will facilitate the development of targeted interventions and strategies aimed at mitigating the adverse impacts of environmental change and ensuring the well-being of affected populations and communities. Meanwhile, simultaneously transcending fixed p-value criteria and incorporating alternative metrics such as effect sizes, confidence intervals, and credibility analysis, in conjunction with the adoption of transparent reporting and sharing practices for data and codes (Matthews 2019), can mitigate publication bias, enhance result interpretation, and provide a robust understanding of the environmental dynamics intrinsic in migration processes.

## Figures and Tables

**Table 1:** Definitions and coding strategies for the covariates in the meta-analysis of environmental migration literature, 2000–2020

	Variable	Definition and coding strategy
Measurements	Migration measures	Migration by whether or not crossing national borders: 1= international migration, 0 = internal migration Migration by direction: 1 = in-migration 2 = net migration 3 = out-migration
	Environmental stressor measures	Environmental stressors by forces: 1 = disaster-related 2 = precipitation-related 3 = temperature-related 4 = land-related 5 = crop/property loss–related 6 = others Environmental stressors by velocities: 1 = rapid-onset, 0 = slow-onset
Study characteristics	Inclusion of control variables	Whether or not control variables are included: 1 = with control variables, 0 = without control variables
	Country	Country types: 1 = non-OECD countries 2 = OECD countries 3 = global
	Dataset time period	Categorial variable for the dataset time period: 1 = dataset from the 1970s 2 = dataset from the 1980s 3 = dataset from the 1990s 4 = dataset from the 2000s 5 = dataset from the 2010s

**Table 2:** Environmental stressors from the selected studies and coding schemes by force and velocity

Environmental stressors	Source	Force	Velocity
Air pollution	Afifi and Warner 2008	Others	Slow onset
Bush fire	Abu, Codjoe, and Sward 2014	Disaster-related	Rapid onset
Climate impact	Nawrotzki and Bakhtsiyarava 2017; Ruyssen and Rayp 2014	Others	Slow onset
Crop yield	Feng, Krueger, and Oppenheimer 2010; Kubik and Maurel 2016	Loss-related	Slow onset
Damaged land	Iqbal and Roy 2015	Land-related	Slow onset
Deforestation	Shrestha and Bhandari 2007; Massey, Axinn, and Ghimire 2010; Shrestha and Bhandari 2007	Land-related	Slow onset
Desertification	Afifi and Warner 2008	Land-related	Slow onset
Drought	Hunter, Murray, and Riosmena 2013; Nawrotzki et al. 2017	Precipitation-related	Slow onset
Earthquake	Bohra-Mishra, Oppenheimer, and Hsiang 2014; Gröschl and Steinwachs 2017	Disaster-related	Rapid onset
El Niño	Curran and Meijer-Irons 2014	Others	Slow onset
Environmental condition	Stojanov et al. 2017; Duda, Fasse, and Grote 2018	Others	Slow onset
Flood	Gray and Mueller 2012; Logan, Issar, and Xu 2016	Precipitation-related	Rapid onset
Humidity	Poston et al. 2009	Others	Slow onset
Hurricane	Chort and Rupelle 2016; Smith and McCarty 2009; Loebach 2016	Disaster-related	Rapid onset
La Niña	Curran and Meijer-Irons 2014	Others	Slow onset
Land erosion	Bernzen, Jenkins, and Braun 2019; Gray 2010	Land-related	Slow onset
Land quality	Gray and Bilsborrow 2013; Gray 2011; Abu, Codjoe, and Sward 2014. 2014	Land-related	Slow onset
Landslide	Bohra-Mishra, Oppenheimer, and Hsiang 2014	Land-related	Rapid onset
Livestock loss	Adoho and Wodon 2014	Loss-related	Slow onset
Monsoon delay	Thiede and Gray 2017	Precipitation-related	Slow onset
NDVI	Bhattacharya and Innes 2008	Others	Slow onset
Property damage	Schultz and Elliott 2013	Loss-related	Rapid onset
Rainfall	Call et al. 2017; Missirian and Schlenker 2017	Precipitation-related	Slow onset
Sea level rise	Afifi and Warner 2008	Temperature-related	Slow onset
Soil degradation	Henry, Boyle, and Lambin 2003	Land-related	Slow onset
Soil pollution	Afifi and Warner 2008	Land-related	Slow onset
Soil salinization	Chen and Mueller 2018	Land-related	Slow onset
SPEI	Abel et al. 2019	Precipitation-related	Slow onset
Storm	Goldbach 2017	Disaster-related	Rapid onset
Temperature	Mueller, Gray, and Kosec 2014; Riosmena, Nawrotzki, and Hunter 2018; Nawrotzki et al. 2015	Temperature-related	Slow onset
Tsunami	Afifi and Warner 2008	Disaster-related	Rapid onset
Volcanic explosion	Gröschl and Steinwachs 2017	Disaster-related	Rapid onset
Weather condition	Adoho and Wodon 2014; Stojanov et al. 2017; Nawrotzki and Bakhtsiyarava 2017; Nguyen and Wodon 2014	Others	Slow onset
Wind	Poston et al. 2009	Others	Slow onset

*Note*: The sources for each environmental measure are not exhaustive; they are typical cases for each environmental measure.

**Table 3:** Descriptive statistics of coded dependent and independent variables from the environmental migration literature, 2000–2020

Variable	N	Mean/percentage
***PCC and associated standard error***		
PCC	3,317	0.01
Standard error of PCC	3,313	0.03
***Migration measures***		
International migration	1,799	53.22%
Internal migration	1,581	46.78%
In-migration	188	5.56%
Net migration	719	21.27%
Out-migration	2,473	73.17%
***Environmental stressor measures***		
Disaster-related	867	25.65%
Precipitation-related	1,191	35.24%
Temperature-related	813	24.05%
Land-related	121	3.58%
Loss-related	317	9.38%
Others	71	2.10%
Slow onset	2,390	70.71%
Rapid onset	990	29.29%
***Study characteristics***		
OECD countries	1,050	31.07%
Non-OECD countries	1,573	46.54%
Global	757	22.40%
With control variables	2,754	81.48%
Without control variables	626	18.52%
Dataset from the 1970s	36	1.07%
Dataset from the 1980s	882	22.09%
Dataset from the 1990s	928	27.46%
Dataset from the 2000s	1,359	40.21%
Dataset from the 2010s	175	5.18%

*Note*: PCCs range from −0.999 to 0.997, and their associated standard errors range from 0.000 to 0.228. We reported percentages for dummy and categorical variables.

**Table 4:** Direction and significance of environment–migration relationship across out-, in-, and net migration, 2000–2020

	Out-migration	In-migration	Net migration
Insignificant	1,521 (61.5%)	56 (29.8%)	461 (64.1%)
Significant	916 (37.0%)	132 (70.2%)	245 (34.1%)
Positive	531 (58.0%)	55 (41.7%)	167 (68.2%)
Negative	385 (42.0%)	77 (58.3%)	78 (31.8%)
Total	2,437	188	706

*Note:* p<0.05 is used to distinguish between significant and insignificant results. Of the total estimates, 49 are missing because they do not report significance levels.

**Table 5:** Weighted averages of the overall partial correlation coefficient and partial correlation coefficient by subgroups, 2000–2020

Environmental stressors	Out-migration	In-migration	Net migration
***Overall***	0.001 (n = 2,473)	−0.288 (n = 188)	−0.189 (n = 719)
***Subgroup by force***			
Disaster-related	0.425 (n = 582)	−0.434 (n = 121)	−0.363 (n = 164)
Land-related	0.007 (n = 114)	N/A	−0.007 (n = 7)
Loss-related	−0.014 (n = 286)	0.070 (n = 24)	0.057 (n = 7)
Precipitation-related	−0.006 (n = 912)	0.051 (n = 27)	0.003 (n = 252)
Temperature-related	0.001 (n = 518)	−0.012 (n = 12)	0.007 (n = 283)
Others	0.008 (n = 61)	0.505 (n = 4)	−0.001 (n = 6)
***Subgroup by velocity***			
Rapid onset	0.031 (n = 674)	−0.434 (n = 123)	−0.330 (n = 193)
Slow onset	−0.003 (n = 1,799)	0.038 (n = 65)	0.005 (n = 526)

*Note:* The precision-based weight was used as weight. The numbers of observations for each category are in parentheses. There are no land-related environmental stressors in studies focusing on in-migration.

**Table 6:** Multivariate meta-regression predicting the partial correlation coefficient of environmental impacts on out- and net migration by environmental force and velocity and other covariates, 2000–2020

	Out-migration				Net migration			
	*OLS*	*IV*	*OLS*	*IV*	*OLS*	*IV*	*OLS*	*IV*
**Environmental stressors by force** (ref. = others)							
Disaster-related	0.00 (0.01) [0.905]	0.28 (0.08) [0.001]			0.06 (0.07) [0.426]	−0.20 (0.05) [0.000]		
Land-related	0.02 (0.02) [0.363]	−0.10 (0.07) [0.155]			0.06 (0.06) [0.297]	−0.03 (0.04) [0.473]		
Loss-related	−0.06 (0.03) [0.091]	0.05 (0.09) [0.600]			0.01 (0.10) [0.910]	−0.00 (0.09) [0.978]		
Precipitation-related	−0.01 (0.01) [0.367]	−0.08 (0.08) [0.275]			0.05 (0.07) [0.430]	0.04 (0.06) [0.434]		
Temperature-related	−0.01 (0.02) [0.726]	−0.08 (0.07) [0.274]			0.08 (0.05) [0.149]	0.05 (0.04) [0.239]		
**Environmental stressors by velocity** (ref. = slow onset)						
Rapid onset			0.01 (0.01) [0.205]	0.01 (0.01) [0.384]			−0.01 (0.02) [0.712]	−0.22 (0.01) [0.000]
**Migration measure** (ref. = internal migration)								
International migration	−0.04 (0.02) [0.027]	−0.29 (0.06) [0.000]	−0.03 (0.01) [0.020]	−0.20 (0.09) [0.019]	−0.03 (0.02) [0.055]	0.10 (0.07) [0.123]	−0.03 (0.02) [0.051]	0.10 (0.08) [0.196]
**Study characteristics**								
OECD countries	−0.02 (0.01) [0.095]	−0.15 (0.03) [0.000]	−0.04 (0.01) [0.001]	−0.19 (0.04) [0.000]	−0.00 (0.02) [0.969]	−0.08 (0.13) [0.540]	−0.01 (0.03) [0.736]	0.14 (0.13) [0.297]
Global	0.00 (0.01) [0.875]	0.22 (0.11) [0.039]	−0.00 (0.01) [0.796]	0.30 (0.13) [0.026]	0.00 (0.01) [0.902]	−0.22 (0.08) [0.003]	0.00 (0.01) [0.930]	−0.22 (0.08) [0.007]
Including controls	0.01 (0.01) [0.338]	−0.02 (0.11) [0.873]	0.02 (0.01) [0.021]	−0.01 (0.10) [0.893]	−0.01 (0.00) [0.020]	−0.21 (0.06) [0.000]	−0.01 (0.00) [0.129]	−0.22 (0.06) [0.000]
Dataset from 1980s	0.02 (0.02) [0.344]	0.05 (0.13) [0.714]	0.01 (0.02) [0.537]	0.13 (0.12) [0.292]	0.06 (0.02) [0.007]	−0.04 (0.06) [0.524]	0.05 (0.02) [0.027]	0.16 (0.06) [0.005]
Dataset from 1990s	−0.01 (0.02) [0.790]	−0.26 (0.02) [0.000]	−0.02 (0.02) [0.348]	−0.26 (0.03) [0.000]	0.07 (0.02) [0.004]	0.20 (0.05) [0.000]	0.06 (0.03) [0.048]	0.44 (0.04) [0.000]
Dataset from 2000s	−0.02 (0.01) [0.126]	−0.13 (0.03) [0.000]	−0.02 (0.01) [0.118]	−0.14 (0.05) [0.002]	0.07 (0.03) [0.050]	−0.02 (0.17) [0.917]	0.05 (0.04) [0.182]	0.20 (0.17) [0.235]
Dataset from 2010s	−0.03 (0.02) [0.090]	0.14 (0.06) [0.020]	−0.03 (0.02) [0.064]	0.09 (0.07) [0.194]	0.04 (0.03) [0.125]	0.07 (0.05) [0.118]	0.04 (0.03) [0.221]	0.13 (0.11) [0.238]
Standard error of the PCC	0.89 (0.42) [0.040]	−8.27 (9.34) [0.376]	0.89 (0.41) [0.038]	−5.11 (9.37) [0.586]	0.81 (0.26) [0.012]	−0.96 (2.10) [0.649]	0.77 (0.33) [0.045]	−0.70 (2.18) [0.749]
Constant	0.03 (0.02) [0.049]	0.36 (0.18) [0.047]	0.02 (0.01) [0.224]	0.28 (0.13) [0.027]	−0.10 (0.04) [0.029]	0.17 (0.18) [0.353]	−0.03 (0.04) [0.521]	−0.01 (0.23) [0.971]
Observations	2,409	2,409	2,409	2,409	719	719	719	719
R-squared	0.11	0.51	0.10	0.44	0.05	0.21	0.04	0.21
AIC	−4,144		−4,105		−882		−878	
BIC	−4,068		−4,053		−841		−841	
F-test for the relevance of the instrument		159		168		3,389		3,209
Wooldridge’s test for exogeneity of the instrument		0.01		0.01		1.89		2.36

*Note*: Standard errors in parentheses; p-values in brackets. For both OLS and IV regressions, the precision-based weight was used as the weight. For IV regression, the inverse of the square root of the number of degrees of freedom was used as the instrument. The reference groups for OECD countries, global (including controls), and dataset are non-OECD countries (without controls) and dataset from the 1970s.

## Footnotes

3 In this paper, we use “study” to refer to each of the individual papers selected for this meta-analysis and use “estimate” to refer to the coefficient of environmental stressors from the individual papers.

4 Net migration is usually calculated by subtracting out-migrants from in-migrants. So increased net migration generally means there are more in-migrants than out-migrants, with decreasing net migration involving the opposite.

5 Stojanov et al. (2014) cautioned that the current typologies of environmental migrants oversimplified the complexity of environmentally induced population movements and that researchers should consider including social, economic, and political contexts from a multidisciplinary approach.

6 In meta-analysis, r-based effect sizes reflect correlations between variables. These values represent the strength and direction of the relationship between an independent variable and a dependent variable. d-based effect sizes, on the other hand, indicate standardized mean differences between groups or categories of variables, offering insights into the magnitude of differences across these groups or categories. Odds-based effect sizes reflect the ratio or odds of outcomes occurring in different groups, particularly in the comparison of two categories across different variables in a meta-analysis setting. These effect sizes are fundamental for aggregating and comparing findings across multiple studies.

7 For instance, if sample size n=200, the number of covariates k=9 (which gives the degrees of freedom of 190, i.e., n−k−1), and the two stars ** representing p<0.05, to get a t value we could let p equal 0.05 and then get the two-tailed t value of 1.97. However, as the true values of p get smaller, t values will increase. Therefore, the t value from this approach represents the lower-bound estimate of the true t values. According to Equation (1), if t values are lower-bound estimates, then PCC is underestimated. In other words, including studies without appropriate statistics for constructing PCC will potentially underestimate the true effect sizes between environmental factors and migration.

8 We included non-anthropogenic or tectonic events such as earthquakes, storms, and hurricanes because those disasters have been shown to trigger migration and/or relocation and because studies related to those events comprise a considerable portion of the environmental migration literature. For example, Hurricane Katrina, one of the deadliest to strike the United States in recent years, has become a symbolic phenomenon in the environmental migration literature and continues to attract the attention of researchers more than 15 years later.

9 The previous literature tends to explore the impact of flood and drought separately. In this study we combine them into precipitation-related stressors because they represent the two extremes of precipitation conditions, with extreme high precipitation causing floods and extreme low precipitation causing drought; both have been shown to be associated with migration.

10 Land-, property-, and crop-related loss could be the results of other environmental stressors, such as hurricanes and floods; therefore they may not be exogeneous variables, causing endogeneity issues in the relationship between them and migration. In this study we treated land- and loss-related stressors as measures of environmental stressors without distinguishing the linkages among environmental stressors, losses, and migration because this is not the focus of this meta-analysis.

11 We acknowledge that significance level alone, especially using an arbitrary criterion such as p<0.05, is not sufficient to claim a significant finding. The significance results listed in Table 4 come from empirical studies where authors usually used the criterion of p<0.05 to distinguish between significant and insignificant results.

12 The finding from the weighted average PCC (Table 5) that environmental stressors generally suppress net migration does not necessarily contradict the finding shown in Table 4, which suggests that the majority (68%) of results from the selected studies in this meta-analysis reported that environmental stressors tended to increase net migration. This is because the analyses from the two tables are conducted at different levels. (Table 5 reported aggregated averages across environment and migration measures, taking into account estimate precision; Table 4 demonstrated the distribution of individual environmental impacts on various migration measures.) Therefore they are not directly comparable, and results shown in the two tables are not necessarily contradictory.

13 Because we use one instrument for one endogenous variable, there is no overidentification problem. Additionally, we calculated a robust variance estimate in the models. Therefore Wooldridge’s score test, instead of the Durbin and Wu-Hausman tests, is reported for the test of the exogeneity of the instrument.

14 The fact that rapid-onset environmental factors are not associated with out-migration is well documented in previous research. The reason could be that rapid-onset environmental factors may quickly deplete financial resources needed to initiate a move elsewhere. And the findings from the multivariate and IV regressions do not necessarily contradict the result from the weighted average PCC (Table 5), showing that rapid-onset environmental stressors are positively associated with out-migration, because the regressions include multiple covariates, whereas the weighted averaged PCC considers only the relationship between rapid-onset environmental stressors and out-migration without controlling for any covariates.

15 Theoretically, the 188 in-migration estimates can be combined with net migration because the expected coefficient directions would be similar for both of them. To test this possibility, we combined in- and net migration and conducted a sensitivity analysis. Table A-2 shows the results. The R-squared value is smaller than that of considering only net migration, and the results differ, especially for international migration, OECD countries, and research datasets from the 2000s. The results suggest that combining in- and net migration does not improve model fitting and that net migration may better be analyzed separately.

16 Again, this conclusion does not inherently conflict with the previous statement that rapid-onset stressors exhibited a more substantial impact on migration. The former conclusion arises from empirical models that assess the importance or significance of these factors; the latter conclusion is derived from the weighted average PCC of rapid-onset environmental stressors at an aggregated level without considering the importance or significance of these factors.

## Appendix A

**Table A-1:** Recently published systematic review on the environmental migration literature

Author(s) and year	Title	Journal	Studies
Obokata, Veronis, and McLeman (2014)	Empirical Research on International Environmental Migration: A Systematic Review	Population and Environment	31
Neumann and Hermans (2017)	What Drives Human Migration in Sahelian Countries? A Meta-analysis	Population, Space and Place	41
Borderon et al. (2019)	Migration Influenced by Environmental Change in Africa: A Systematic Review of Empirical Evidence	Demographic Research	53
Hoffmann et al. (2020)	A Meta-analysis of Country-Level Studies on Environmental Change and Migration	Nature Climate Change	30
Kaczan and Orgill-Meyer (2020)	The Impact of Climate Change on Migration: A Synthesis of Recent Empirical Insights	Climatic Change	17
Beine and eusette (2021)	A Meta-analysis of the Literature on Climate Change and Migration	Journal of Demographic Economics	51
Barbora Šedová, Čizmaziová, and Cook (2021)	A Meta-analysis of Climate Migration Literature	Center for Economic Policy Analysis (CEPA) Discussion Papers	116

**Table A-2:** Sensitivity analysis by combining in- and net migration

	OLS	IV	OLS	IV
**Environmental stressors by force** (ref. = others)				
Disaster-related	−0.06 (0.06) [0.336]	−0.24 (0.08) [0.003]		
Land-related	−0.04 (0.06) [0.539]	−0.06 (0.06) [0.308]		
Loss-related	−0.05 (0.07) [0.489]	0.05 (0.09) [0.538]		
Precipitation-related	−0.05 (0.07) [0.452]	0.04 (0.06) [0.512]		
Temperature-related	−0.03 (0.07) [0.660]	0.02 (0.07) [0.813]		
**Environmental stressors by velocity** (ref. = slow onset)				
Rapid onset			−0.02 (0.02) [0.396]	−0.24 (0.02) [0.000]
**Migration measure** (ref. = internal migration)				
International migration	−0.05 (0.03) [0.127]	0.42 (0.10) [0.000]	−0.04 (0.03) [0.224]	0.43 (0.10) [0.000]
Migration measure (ref. = internal migration)				
**Study characteristics**				
OECD countries	−0.07 (0.03) [0.017]	0.16 (0.03) [0.000]	−0.06 (0.03) [0.045]	0.40 (0.05) [0.000]
Global	−0.01 (0.01) [0.418]	−0.32 (0.09) [0.000]	−0.01 (0.01) [0.498]	−0.33 (0.09) [0.000]
Including controls	−0.01 (0.02) [0.755]	−0.20 (0.08) [0.016]	−0.01 (0.02) [0.757]	−0.21 (0.07) [0.004]
Dataset from 1980s	0.00 (0.02) [0.840]	−0.16 (0.09) [0.074]	0.01 (0.02) [0.555]	0.07 (0.07) [0.306]
Dataset from 1990s	0.01 (0.03) [0.687]	0.10 (0.09) [0.278]	0.02 (0.03) [0.437]	0.36 (0.07) [0.000]
Dataset from 2000s	−0.02 (0.05) [0.724]	0.13 (0.07) [0.058]	−0.00 (0.05) [0.927]	0.39 (0.05) [0.000]
Dataset from 2010s	0.02 (0.05) [0.678]	0.13 (0.07) [0.066]	0.02 (0.05) [0.599]	0.20 (0.14) [0.169]
Standard error of the PCC	0.65 (0.52) [0.231]	−5.30 (3.34) [0.113]	0.72 (0.51) [0.184]	−5.25 (3.17) [0.098]
Constant	0.08 (0.10) [0.405]	0.08 (0.09) [0.348]	0.03 (0.04) [0.547]	−0.13 (0.10) [0.175]
Observations	904	904	904	904
R-squared	0.04	0.17	0.04	0.17
AIC	−750		−753	
BIC	−693		−710	
F-test for the relevance of the instrument		248		304
Wooldridge’s score test for exogeneity of the instrument		0.17		0.17

*Note:* Standard errors in parentheses; p-value in brackets. For both OLS and IV regressions, the precision-based weight was used as the weight. For IV regression, the inverse of the square root of the number of degrees of freedom was used as the instrument. The reference groups for OECD countries, global (including controls), and dataset are non-OECD countries (without controls) and dataset from the 1970s.

**Table A-3:** Sensitivity check predicting the partial correlation coefficient of environmental impacts on out- and net migration by environmental force and velocity and other covariates using two-level regression method, 2000–2020

	Out-migration		Net migration	
**Level-1 characteristics**				
Disaster-related	−0.04 (0.02) [0.047]		0.06 (0.06) [0.278]	
Land-related	−0.03 (0.02) [0.278]		0.06 (0.07) [0.389]	
Loss-related	−0.06 (0.03) [0.024]		0.01 (0.08) [0.874]	
Precipitation-related	−0.05 (0.02) [0.025]		0.05 (0.06) [0.330]	
Temperature-related	−0.04 (0.02) [0.072]		0.08 (0.06) [0.164]	
Rapid onset		0.00 (0.01) [0.620]		−0.01 (0.01) [0.528]
International migration	−0.00 (0.01) [0.982]	−0.00 (0.01) [0.966]	−0.03 (0.04) [0.338]	−0.03 (0.03) [0.318]
**Level-2 characteristics**				
OECD countries	−0.04 (0.02) [0.059]	−0.05 (0.02) [0.030]	−0.00 (0.04) [0.982]	−0.01 (0.04) [0.801]
Global	−0.05 (0.03) [0.094]	−0.05 (0.03) [0.076]	0.00 (0.01) [0.915]	0.00 (0.01) [0.905]
Including controls	−0.00 (0.02) [0.989]	0.00 (0.02) [0.824]	−0.01 (0.02) [0.631]	−0.01 (0.02) [0.752]
Dataset from 1980s	0.01 (0.10) [0.900]	0.01 (0.10) [0.896]	0.06 (0.04) [0.095]	0.05 (0.04) [0.164]
Dataset from 1990s	−0.03 (0.10) [0.772]	−0.03 (0.10) [0.782]	0.07 (0.04) [0.086]	0.06 (0.04) [0.131]
Dataset from 2000s	−0.03 (0.10) [0.715]	−0.03 (0.10) [0.715]	0.07 (0.06) [0.221]	0.05 (0.06) [0.327]
Dataset from 2010s	−0.01 (0.10) [0.932]	0.00 (0.10) [0.998]	0.04 (0.04) [0.280]	0.04 (0.04) [0.337]
Standard error of the PCC	0.78 (0.19) [0.000]	0.77 (0.19) [0.000]	0.81 (0.26) [0.002]	0.76 (0.27) [0.005]
Constant	0.09 (0.10) [0.365]	0.05 (0.10) [0.636]	−0.10 (0.08) [0.199]	−0.03 (0.05) [0.592]
Observations	2,409	2,409	719	719
Number of studies	105	105	24	24
AIC	−4,561	−4,559	−868	−868
BIC	−4,463	−4,484	−794	−808
ICC	0.507	0.505	0.000	0.001

*Note:* Standard errors in parentheses; p-value in brackets. Level-1 characteristics encompass measurements of environmental stressors and migration. Level-2 characteristics consist of higher-level study-specific measures, such as the time, location, and methodology of the research. The reference groups for OECD countries, global (including controls), and dataset are non-OECD countries (without control) and dataset from the 1970s.

## Appendix B. Literature filtering processes

Once the literature was collected, we dropped the duplicates and then applied two rounds of filtering. In the first round, we screened the literature by title and abstract, and applied the following criteria in excluding literature:

1. Studies not written in English. This criterion excluded 124 studies, most of which were written in French (66.94%), Spanish (15.32%), and Portuguese (11.29%). Those studies were excluded because we are currently unable to process non-English languages.
2. Studies published before 2000. This criterion excluded 37 studies that were primarily introductions to environmental migration (e.g., Westing 1992; Wolpert 1966) and case studies explaining various environmental stressors and involved populations (e.g., Bilsborrow 1992). These types of studies are too descriptive and often unable to provide sufficient statistics for a quantitative meta-analysis.
3. Studies that are reports, summaries, qualitative research, or irrelevant to environmental migration. This criterion excluded 859 studies. Some studies are reports and summaries (e.g., Deheza and Mora 2011) and qualitative research (e.g., Doevenspeck 2011). As mentioned above, the employment of PCC requires correlation matrices or regression results at a minimum; therefore studies that use only descriptive statistics or qualitative data were omitted. Others are studies on human rights and justice for environmental migrants and refugees (e.g., Dreher and Voyer 2015) and are not focused on environmental migration.^17^

After the first round of filtering, we had 478 studies. In the second round of filtering, we screened the 478 studies by reading the full text and excluded those that did not include environmental stressors or included only summaries and descriptive statistics. The second filtering excluded 349 studies, leaving us with 128 studies for the meta-analysis. Each study may include multiple environmental stressors and multiple regression models, therefore providing more estimates than the number of studies.^18^ In total, from the 128 studies, we retrieved 3,380 estimates of environmental effects on migration.
